# Cost-Benefit Analysis of Preventing Acute Care Use in Oncology Patients Following Systemic Therapy Using Medicare Claims Data: Retrospective Cohort Study

**DOI:** 10.2196/77891

**Published:** 2025-12-11

**Authors:** Sara Alessandra Keller, Maximilian Schuessler, Behzad Naderalvojoud, Tina Seto, Lu Tian, Mohana Roy, Tina Hernandez-Boussard

**Affiliations:** 1 Division of Computational Medicine, Stanford University, 1265 Welch Rd, Stanford, CA, 94305-5479, United States, 1 650-725-5507; 2 Department of Health Sciences and Technology, ETH Zurich, Zurich, Switzerland; 3 Department of Biomedical Data Science, Stanford University, Stanford, CA, United States; 4 Department of Technology & Digital Solutions, Stanford Health Care, Stanford, CA, United States; 5 Department of Medicine, Stanford University School of Medicine and Stanford Cancer Institute, Stanford, CA, United States; 6 Department of Medicine, Med/Oncology, Stanford University, Stanford, CA, United States

**Keywords:** patient care, evidence-based emergency medicine, machine learning, electronic health records, cost-benefit analysis, cost savings, emergency service, oncology service, preventive services, Medicare

## Abstract

**Background:**

Acute care use (ACU) represents a major economic burden in oncology, which can ideally be prevented. Existing models effectively predict such events.

**Objective:**

We aimed to quantify the cost savings achieved by implementing a model to predict ACU in oncology patients undergoing systemic therapy.

**Methods:**

This retrospective cohort study analyzed patients with cancer at an academic medical center from 2010 to 2022. We included patients who received systemic therapy and identified ACU events occurring after treatment initiation, excluding those with known death dates within the study period. Data on ACU-related expenses were gathered from Medicare claims and mapped to service codes in electronic health records, yielding average daily costs for each patient over 180 days following the start of therapy. The exposure was an ACU event.

**Results:**

The main outcome was the average daily cost per patient at the end of the first 180 days of systemic therapy. We observed that expense accumulation flattened earlier and more rapidly among non-ACU patients. This study included 20,556 patients, of whom 3820 (18.58%) experienced at least 1 ACU. The average daily cost per patient for those with and without ACU was US $94.62 (SD US $72.54; 95% CI US $92.32-$96.92) and US $53.28 (SD US $59.92; 95% CI US $52.37-$54.19), respectively. The average total cost per ACU and non-ACU patient was US $17,031.92 (SD US $13,056.63; 95% CI US $16,616.74-$17,445.09) and US $9591.06 (SD US $10,785.83; 95% CI US $9427.64-$9754.48), respectively. To estimate the long-term financial impact of deploying the predictive model, we conducted a cost-benefit analysis based on an annual cohort size of 2177 patients. In the first year alone, the model yielded projected savings of US $910,000. By year 6, projected savings grew to US $9.46 million annually. The cumulative avoided costs over a 6-year deployment period totaled approximately US $31.11 million. These estimates compared the baseline cost model to the intervention model assuming a prevention rate of 35% for preventable ACU events and an average ACU cost of US $17,031.92 (SD US $13,037).

**Conclusions:**

Predictive analytics can significantly reduce costs associated with ACU events, enhancing economic efficiency in cancer care. Further research is needed to explore potential health benefits.

## Introduction

### Acute Care Use Burden in Oncology

Systemic therapy, such as chemotherapy, while essential for treating cancer, can be highly toxic, often leading to complications that result in patients requiring acute care use (ACU), including unplanned hospitalizations and emergency department visits [1]. These events can be classified as avoidable or unavoidable based on clinical context, with avoidable ACU events being potentially preventable through prompt interventions. ACU events are linked to higher rates of repeat visits and mortality [2], and there is evidence suggesting that up to 35% of ACU events are preventable within 180 days of therapy start [3], whereas other sources even discuss possible prevention from 40% to 67% [4-6]. For that reason, these events not only are detrimental to patient health but also significantly contribute to health care costs. Therefore, the Centers for Medicare and Medicaid Services (CMS) has named certain ACU events as preventable and has begun using them as quality metrics, as seen with the Outpatient Quality Reporting Program and the OP-35 measure [7]. The potential to reduce preventable ACU events presents an important opportunity to improve patient outcomes and lower costs for both hospitals and patients.

### Predictive Advancements

Recent advancements in machine learning (ML) have greatly improved the identification of patients at elevated risk of ACU events [1]. Innovations in deep learning and natural language processing now enable more accurate and reliable predictive models, supporting precise risk stratification while remaining robust across diverse populations and periods [8-10]. This capacity creates a paradigm-shifting opportunity; by predicting ACU events before they occur, these models empower health care systems to shift from reactive to proactive care. These electronic health record (EHR)–derived risk scores allow clinicians to deliver targeted interventions for patients at high risk of ACU and potentially prevent costly ACU events.

However, realizing this potential requires navigating significant challenges [11]. This includes the need for dedicated staffing, regulatory compliance, and integration with existing systems, all of which incur significant costs that must be considered in return-on-investment (ROI) assessments. Moreover, successful implementation depends on both technical execution and clinical adoption [11]. Literature on model deployment challenges, clinical integration, and operational cost considerations shows that algorithms fail in practice due to workflow misalignment, lack of interpretability, and the hidden resource burden of sustained use [11-13]. Model transparency and reporting frameworks are increasingly critical for building clinician trust as they document intended use cases, performance characteristics across subpopulations, and potential biases, which are all essential considerations when deploying predictive models in health care settings [10]. The deployment of ML models in health care incurs significant costs related to data preparation, regulatory compliance, integration with existing systems, and ongoing maintenance and monitoring [12,14]. Additionally, deploying these models requires an investment in workforce training and infrastructure, making it a costly endeavor [13]. Therefore, despite the promise of ML in health care, the cost of its deployment must be weighed against the savings it generates, which is a complex and often overlooked aspect of deployment in health care. The deployment of ML models in clinical settings presents challenges beyond technical performance [11]. Equally critical for clinical adoption are workflow optimization, staff training, and clinician trust [13]. This trust must be built through transparent tools such as model cards [10,15] that document performance and potential biases across diverse patient populations. Accounting for these demands is essential as their cumulative cost can undermine the financial viability of even the most promising models.

### Bridging the Gap

To address this gap, we developed a cost analysis to assess the expenses associated with deploying an ML model and the potential savings from preventing ACU events among patients with cancer starting treatment at a comprehensive cancer center (CCC). Our hypothesis was that implementing an ML model would lead to cost savings by preventing ACU events and reducing hospital costs. This study aimed to fill a critical gap in the literature [13,16-18] about the ROI for ML deployment in health care, particularly in oncology. This work paves the way for future studies to explore the broader health benefits, potentially driving the adoption of ML in various areas of patient care. Unlike previous studies focused primarily on model performance, this analysis evaluated real-world financial return, offering critical insights for health care systems considering predictive model deployment.

## Methods

### Study Design

This retrospective cohort study used structured EHR data from routine care from a CCC that includes an academic hospital, a community hospital, and a health care alliance. This study provides a cost analysis comparing daily patient costs with and without a prediction model for preventing ACU admissions. A cost-benefit analysis was conducted from the Medicare payer perspective comparing usual care to an intervention involving predictive model deployment. Costs, including staffing and IT infrastructure, were estimated using institutional records and published benchmarks [13,16,19]. We modeled the deployment of a prediction system using standard cost estimates for artificial intelligence tools (see Multimedia Appendix 1). Savings were derived by applying the preventable ACU proportion to the total cohort and the associated cost differential. Claims data mapped to EHR service codes were used to estimate care costs over the 180-day posttherapy window.

### Ethical Considerations

This retrospective analysis was approved by the Stanford University Institutional Review Board (protocol 47644), with a waiver of informed consent due to secondary use of existing records. This research also used publicly available Medicare claims data. All procedures complied with institutional policies, the Declaration of Helsinki, and applicable privacy regulations. In addition, our study adheres to the Minimum Information for Medical Artificial Intelligence Reporting guidelines [15].

### Data Preparation

We calculated costs using a combination of Medicare Physician Fee Schedule (MPFS) [19,20] and average sales price (ASP) [21] data following the CMS methodology and previous published research [16,22,23] (see Table S1 and equations 1-7 in Multimedia Appendix 1). MPFS values were determined based on 3 components: relative value units assigned to each service, a geographic practice cost index to account for regional cost variations, and a conversion factor that translates relative value units into dollar amounts. ASP values were calculated as 106% of the ASP, reflecting the CMS payment limit for drugs and biologicals. For each Healthcare Common Procedure Coding System (HCPCS) code each year and quarter, we summed the MPFS and ASP values to produce a combined fee for a given service at a specific point in time (equation 3 in Multimedia Appendix 1) [23].

### Operational Implementation

The rationale behind the algorithm is that patients predicted to be at high risk of ACU events will be flagged using pop-up notifications (as shown in Figure S1 in Multimedia Appendix 1). To estimate deployment costs within our cost model, we incorporated key operational needs for a predictive model in outpatient oncology care. These included infrastructure setup, model validation, workflow integration, maintenance, and staffing needs. Deployment costs were the largest contributor and encompassed data infrastructure, integration with the Epic EHR system, and real-time workflow design [12,13,24]. Annual maintenance costs comprised technical audits, software updates, and training. We assumed an initial onboarding session per health care provider to cover alert interpretation, risk stratification logic, and escalation protocols. Although the system is not yet deployed, this assumption reflects institutional norms and previous implementations. Ongoing training for new staff and protocol updates were included in maintenance costs. To operationalize the intervention, we included staffing of 0.25 full-time equivalent (FTE) for a registered nurse, who triages alerts and provides patient education. Published data on remote symptom alert triage in oncology indicate that time per alert ranges from under 5 to 10 minutes, with 52% resolved in under 5 minutes and 36% resolved in 5 to 10 minutes [25]. In addition, 0.5 FTE were estimated for an advanced practice provider (APP), who coordinates clinical follow-up with primary oncology teams [3,23]. All operational assumptions were informed by published oncology implementation studies [13,24,26], internal clinical workflows, and expert discussions. A detailed visualization of the modeled deployment workflow is provided in Figure S2 in Multimedia Appendix 1.

### Estimating Costs

To quantify the cost of care for a patient, we mapped MPFS and ASP values to each patient’s services using HCPCS codes [23], including level 1 codes corresponding to Current Procedural Terminology (CPT) codes from the American Medical Association. Service codes and dates were linked to these combined fees to compute the total cost incurred by each patient on each day of the observation period. These daily costs were then aggregated over the therapy period to calculate the total cost per patient per period (TCPP), representing the full cost of services and drugs received by an individual during the entire treatment window. To standardize this measure and enable comparisons across patients with different observation lengths, we derived the cost of care per patient per day (CCPD) by dividing each patient’s TCPP by the number of days in their observation period. Thus, the CCPD represents an average daily cost across the full treatment window, distinct from the raw daily totals. Variable definitions are provided in Table S4 in the Multimedia Appendix 1, with patient-level CCPD values summarized in Table S4 in Multimedia Appendix 1. To estimate the annual cost of ACU before deploying (without) a predictive model, we multiplied the total patient population by the baseline ACU prevalence to obtain the expected number of ACU events (*N_acu,0_*). This value was then multiplied by the average cost per ACU case (*C_acu_*), resulting in an overall cost estimate in the absence of intervention. We developed the following formula for the total cost estimation without a prediction model (*C_0_*; equation 1; Multimedia Appendix 1):


(1) $$C_{0}(X)=C_{acu}\cdot N_{acu,0}\cdot X$$

To assess the cost with a deployed predictive model, we included the model’s implementation cost (*C_deploy_*); ongoing annual maintenance (*C_annual_*); residual ACU costs after prevention efforts (*C_acu,1_*); and additional staffing required for proactive care, including cost of nurses (*C_nurse_*) and APPs (*C_app_*) [16,17]. We estimated the number of preventable ACU events and calculated the proportion of those correctly identified by the model. We also accounted for false positives, patients incorrectly flagged as high risk, and their implications. The false positives were obtained by first calculating how many patients were not at risk of an ACU event and, among these, identifying how many were incorrectly predicted as being at risk. Accordingly, we developed the following formula for the total cost calculation with a prediction model (*C_1_*; equation 21 in Multimedia Appendix 1):


(2) $$\begin{array}{l} C_{1}(x)=C_{deploy}+C_{annual}\;\;\cdot (x-1)\\ +C_{acu,1}\;\;\cdot x+C_{nurse}\;\;\cdot x+C_{app}\;\;\cdot x \end{array}$$

Service costs were derived as described previously [23] by mapping MPFS and ASP values to each patient’s clinical activity using HCPCS and CPT codes. They were then linked by date and aggregated to compute the TCPP and CCPD as outlined previously. HCPCS and CPT codes were grouped into service categories using CMS classification files. We included professional fees via MPFS and ASP values for drug-related codes. Facility fees, patient cost sharing, and contractual adjustments were excluded. The cost models in the detail section in Multimedia Appendix 1 provide more details of the cost models generated with and without a predictive model. Codes were mapped to the cost on CMS-allowed amounts for professional services and drugs. Facility and patient-specific fees were excluded. Figure S6 in Multimedia Appendix 1 shows the most common and most expensive services observed in the cohort.

### Data Analysis

A cost-benefit analysis was conducted to compare the total costs of ACU admissions with and without the use of a prediction model based on the estimated number of preventable events reported in the literature [27]. The total cost without the model was calculated by multiplying the prevalence of ACU cases by the total number of patients and the cost per ACU case. The cost associated with the model includes deployment costs, annual maintenance, and the additional personnel required to interpret predictions and conduct patient outreach (eg, nurses and APPs). The model’s impact on cost reduction was evaluated by estimating the number of preventable ACU cases and extrapolating the potential savings from excluding these cases. We assumed a conservative lower-bound prevention rate of 35% based on an estimate of the proportion of ACU events deemed preventable in the published literature [3,4], which aligns with the minimal threshold observed across multiple oncology care studies. We then applied this rate to the number of high-risk patients identified by the model. Data analyses were conducted using Python (Python Software Foundation) and libraries such as Seaborn and Matplotlib for visualization and SciPy for statistical testing. A Mann-Whitney *U* test was used for nonnormal data distributions with an α level of .05. Chi-square tests were conducted to assess differences in features within the cohort, and 2-tailed *t* tests were applied to evaluate cost differences at various time points (30, 90, and 180 days; Table S1 in Multimedia Appendix 1). A paired *t* test was used to analyze the monetary reduction between the *C_0_* cost model without and the *C*_*1*_ cost model with deployment over the first 6 years of implementation comparing the means of the paired samples.

### Sensitivity Analysis

To evaluate the robustness of our cost-benefit analysis, we conducted a deterministic break-even sensitivity analysis of the ML model and the cost model. Full formulae and constants can be found in Multimedia Appendix 1. For the ML model, the analysis identified threshold values of key model parameters, namely, the sensitivity of the prediction model (*R*_*sens*_), the prevalence of ACU events (*p*_*prev*_), and the total patient volume (*N*_*total*_) that would result in an ROI of ≥0. Each parameter was varied independently while holding the others constant. We defined the cost without a prediction model (*C₀*) as the product of the average ACU cost and the expected number of patients with ACU events. The cost with a prediction model (*C₁*) included fixed deployment costs, annual maintenance costs, reduced ACU burden due to model sensitivity and prevention rate (*p*_*pred*_), and additional costs. We defined *p*_*pred*_ as the proportion of preventable ACU events. For the cost model sensitivity, we conducted a one-way sensitivity test on *p*_*pred*_ keeping the other variables constant.

## Results

### Study Cohort

**Figure 1.**
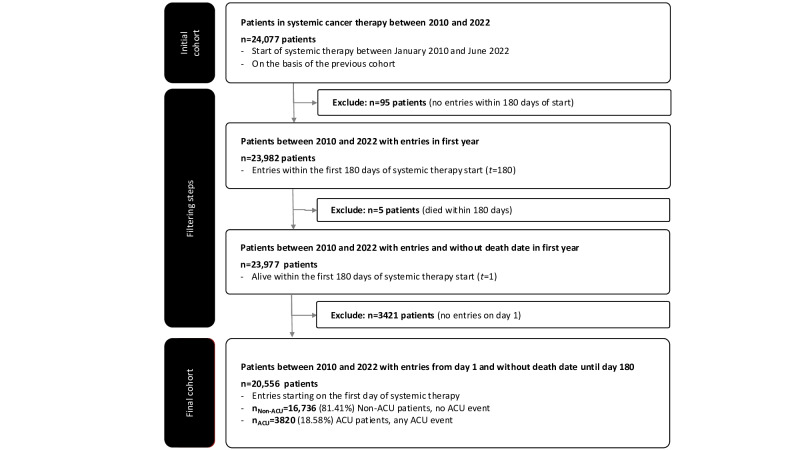
Cohort selection process for cost analysis: flowchart outlining the cohort selection and filtering process for patients undergoing systemic cancer therapy from 2010 to 2022. The initial cohort of 24,077 patients [16] was narrowed down to 20,556 (85.38%) after exclusions based on entry timing, survival within 180 days (approximately 6 months), and missing day 1 records. The final cohort of 20,556 patients included 16,736 (81.42%) non–acute care use (ACU) patients and 3820 (18.58%) ACU patients.

The cohort comprised oncology patients at the CCC who received systemic therapy from 2010 to 2022. Data from the first day of therapy until 180 days after therapy initiation were included, with features such as patient demographics, clinical information, and health services received. Patients without entries within the first year of therapy, those who died within this period, and those without data on the first day of therapy were excluded (Figure 1). Patients were categorized into 2 groups: those with and without ACU admissions, labeled using a binary classification to show whether an ACU admission occurred [10].

The cohort included 20,556 patients, of whom 16,736 (81.42%) had no ACU events and 3820 (18.58%) had at least 1 ACU event, which we defined as the ACU prevalence (Figure 1). Characteristics by ACU status are presented in Table S3 in Multimedia Appendix 1. Differences across demographic and clinical factors were observed in this cohort, including gender, ethnicity, race, insurance type, tumor stage, and tumor type (all *P*<.05; see Table S3 in Multimedia Appendix 1). While several comparisons were statistically significant, we also considered their clinical relevance, as disparities in ACU incidence across demographic groups may reflect differences in acces to care, quality of treatment, or underlying health status. For example, the ACU rate in genitourinary cancer was 42% higher than in breast cancer (23.1% vs 16.2%). Higher ACU proportions were observed for Medicaid coverage (29%; *P*<.001), advanced tumor stage (stage 4: 30%; *P*<.001; stage 3: 22%; *P*=.002), and tumor types including genitourinary (35%; *P*<.001), pancreas (33%; *P*<.001), sarcoma (33%; *P*<.001), and lymph (28%; *P*<.001), whereas breast cancer showed a lower ACU proportion (10%; *P*<.001). Differences in race were also observed between Asian (20%; *P*=.001), Black (23%; *P*=.009), and Hawaiian or Pacific Islander patients (27%, *P*=.002). These findings are descriptive and indicate associations within this cohort rather than causal effects. Patients with ACU events incurred significantly higher costs (*P*<.001), including a TCPP of US $17,031.92 within the first 180 days (approximately 6 months) compared to US $9591.06 for those without ACU events. Daily costs were double for ACU patients (US $94.62 vs US $53.28, respectively; Table S4 in Multimedia Appendix 1).

### Cost per Day

Cost analysis revealed that CCPD and TCPP differed significantly between ACU and non-ACU patients (*P*<.001), with CCPD decreasing over time for all patients but total expenses rising for those with ACU events. At 180 days, the average CCPD in the ACU group was nearly double that of non-ACU patients (US $94.62, SD US $72.54 vs US $53.28, SD US $59.92), respectively; *P*<.001; Table S4 in Multimedia Appendix 1). The higher costs in ACU patients were driven by greater frequency of pathology, laboratory, and medicine-related procedures (Figure S8 and S9 in Multimedia Appendix 1). The cost differences between groups were both statistically and clinically significant (*P*<.001), with ACU patients incurring approximately 78% higher total and daily costs over 180 days than non-ACU patients (US $17,031.92, SD US $13,056.63) vs US $9591.06, SD US $10,785.83, respectively; US $94.62, SD US $72.54 per day vs US $53.28, SD US $ 59.92 per day, respectively).

### TCPP Results

**Figure 2. F2:**
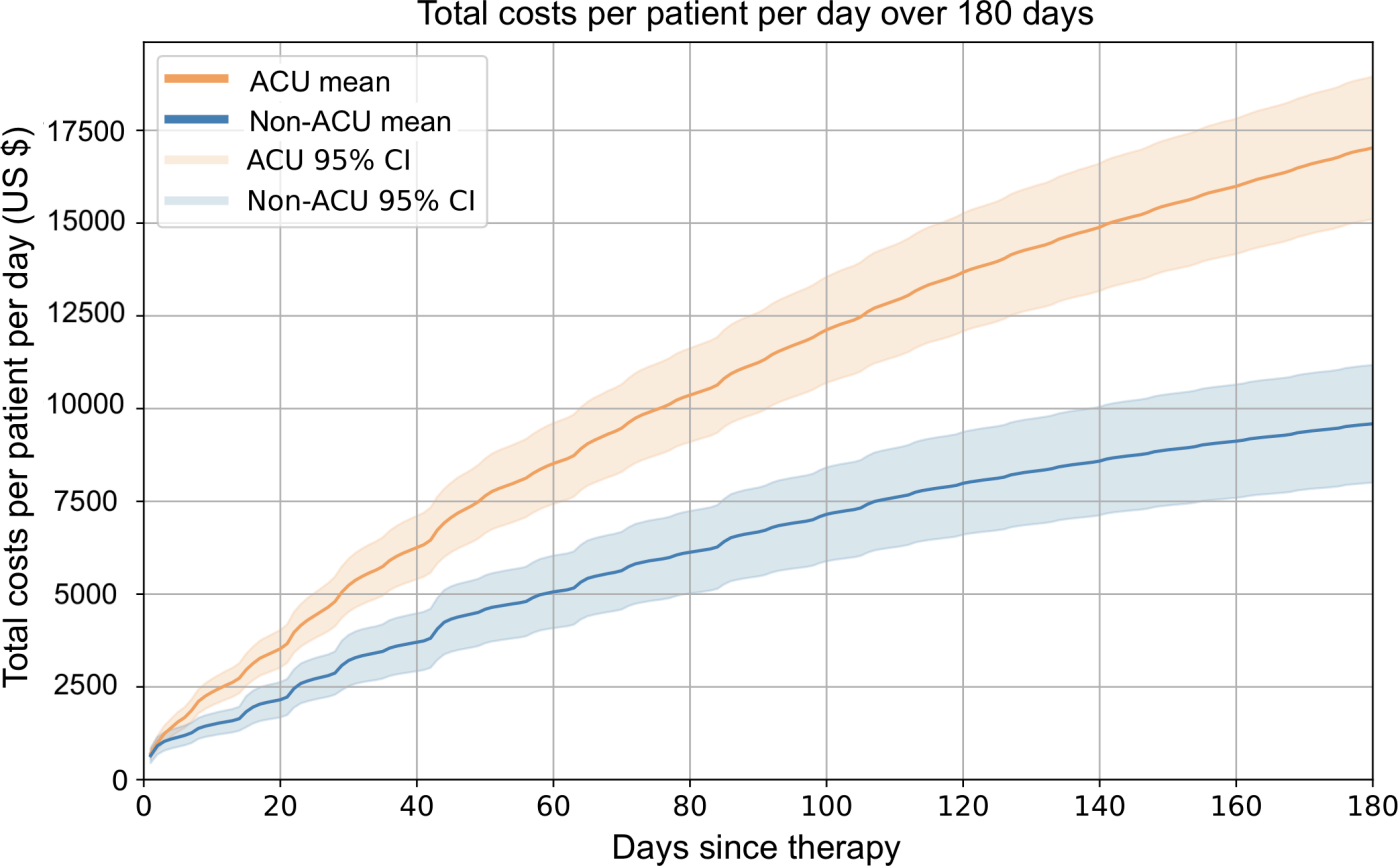
Total costs per patient over 180 days with and without acute care use (ACU) events. The mean total costs per patient per day (US $) over 180 days are presented for patients with ACU events (n=3820; orange line) and those without ACU events (n=16,736; blue line). The shaded areas represent the 95% CIs. Patients experiencing ACU events incurred consistently higher costs throughout the 180-day period.

The average TCPP for patients with ACU within the first 180 days was US $17,031.92 (SD US $13,056.63) compared to US $9591.06 (SD US $10,785.83) for those without such events (Figure 2 [Fig F2]and Figure S5 in Multimedia Appendix 1 ). We observed that expense accumulation flattened earlier and more rapidly for non-ACU patients (*P*<.001). The shaded areas illustrate the variation of the total costs from the mean. On day 180, the average daily cost for those with ACU was US $94.62 (SD US $72.54) compared to US $53.28 (SD US $59.92) for those without ACU, which means that patients with ACU events incurred twice the daily costs (*P*<.001) (Figure 2 and Figure S6 in Multimedia Appendix 1).

### Cost Model Results

**Figure 3.**
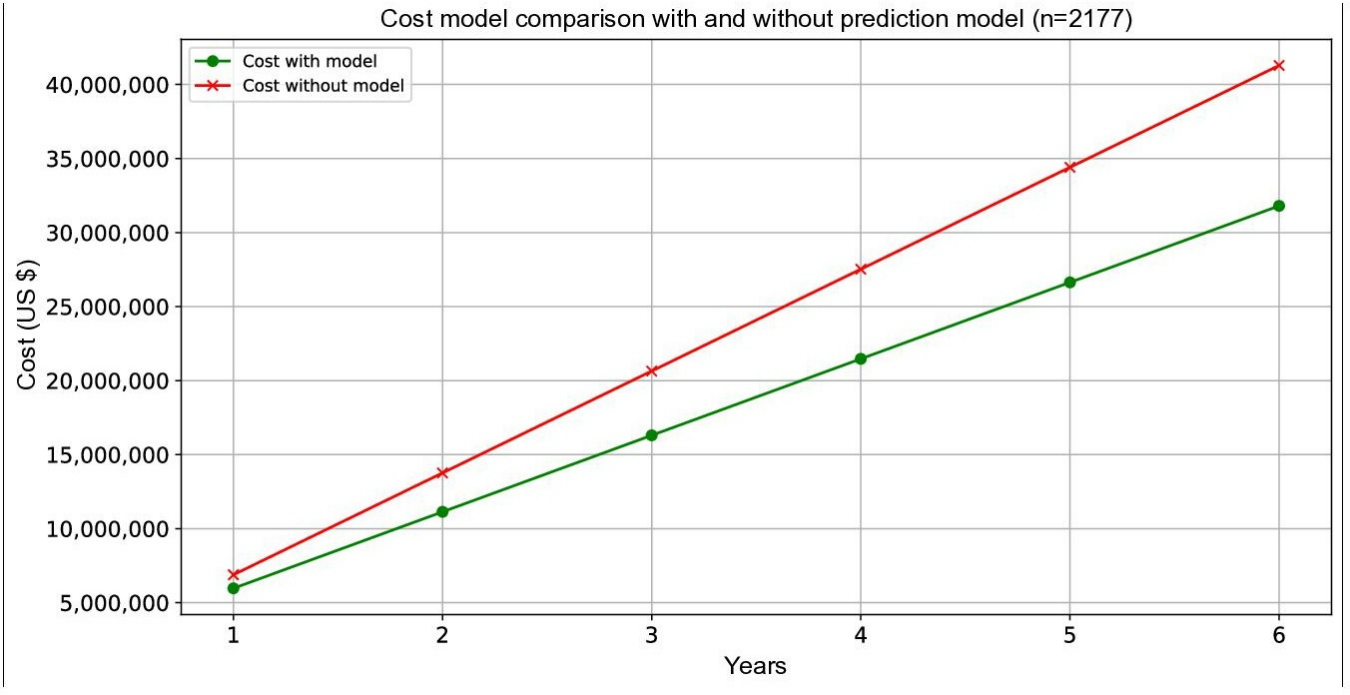
Projected cost comparison with and without the prediction model. Cumulative costs (US $) of patient management are shown over 6 years for a cohort of 2177 patients. Costs with the prediction model (*C_1_*; green line) showed large savings compared to costs without the model (*C_0_*; red line), with cumulative savings of US $31.11 million after accounting for the implementation and operational costs of the predictive model. The prediction model showed a clear economic advantage in managing long-term expenses.

Operational requirements for implementing the predictive model included supplementary staffing of 0.25 FTE nurses and 0.5 FTE APPs, with combined annual staffing costs of US $112,765. Additional fixed costs of model deployment included software integration, staff training, and compliance with health care IT standards, totaling US $1 million in year 1, with US $200,000 in annual maintenance thereafter. The model showed a positive ROI starting in year 1 (US $910,000) and cumulative savings of US $9.46 million by year 6 (Figure 3). If higher prevention rates of up to 64% are achieved, as suggested in the literature, cost savings could increase further. ROI was calculated as net savings divided by total implementation cost, with break-even achieved within the first year. We used a least absolute shrinkage and selection operator model with a sensitivity of 0.84 and specificity of 0.51 to predict ACU events (Figure S7 in Multimedia Appendix 1), identifying 1.65% (339/20,556) of the patients in the cohort as high risk, enabling targeted intervention planning. As mentioned previously, we assumed that 35% of ACU events were preventable as this value represents a conservative lower bound supported by published literature [3,4]. Using this number in our model, we obtained 119 yearly prevented events and projected cost savings of US $31.11 million over 6 years in our cohort. We expected a cost saving of approximately US $1 million in the year of deployment. There is potential for greater impact as we used a prevention rate of 35% and different prevention rates have been observed in different studies (between 35% and 67%) [4-6]. In contrast to the annualized economic analysis, we estimate with the ACU rate of 18.58% (3820/20,556) in a similar sized cohort, that the number of patients experiencing ACU events over 6 years would be reduced from 8484 to 5985, resulting in 2499 fewer ACU-affected patients. This reduction in avoidable acute care episodes underscores the clinical benefit of early triage and intervention.

### Sensitivity Analysis

The model reached break-even within 6 years if the prevention rate was higher than 27%. Prevention rates of 35% or higher [4-6] consistently led to early cost savings within the first year of deployment. Lower-effectiveness scenarios did not achieve break even within the evaluation window (see the sensitivity analysis section in Multimedia Appendix 1 and Figure S10 in Multimedia Appendix 1).

## Discussion

### Principal Findings

#### Overview

In this retrospective study, we explored the economic impact of ACU in oncology patients starting chemotherapy and the cost benefits of implementing a predictive model to identify and intervene on high-risk patients. Our analysis of over 20,000 patients initiating chemotherapy revealed that ACU events nearly double daily costs, significantly burdening both patients and the health care system. By implementing an ML model to identify patients at high risk of ACU, we observed substantial cost reductions and a favorable ROI for the health care system. These findings underscore the importance of proactive interventions in oncology, demonstrating that predictive analytics and digital tools can enhance patient management, improve outcomes, and address health care inequities.

#### Economic Burden of ACU Events

The cost implications of ACU events within our cohort were significant, with patients experiencing ACU incurring an average total cost per patient of US $17,031.92 in the first 180 days of treatment compared to just US $9591.06 for those without ACU events (*P*<.001). The 77.5% higher total costs for ACU patients were not just statistically significant but also clinically meaningful, directly impacting both health care budgets and patient quality of life. This stark contrast in costs between patient groups reinforces previous research that underscores the considerable financial burden that ACU places on both patients and health care systems [16]. Furthermore, we observed that the cumulative financial impact of ACU events increased over time, further straining health care resources, as noted by Iqbal [27] and Oluwagbade and Covenant [28]. The notable differences in CCPD between the 2 groups emphasize the critical importance of early identification of high-risk patients to mitigate unnecessary health care expenditures. This notion aligns with existing literature that advocates for early risk identification and preventive interventions, which can lead to more efficient health care resource allocation [29].

#### Cost Savings From Preventive Intervention

This study highlights that deploying a predictive model is an effective strategy for reducing preventable ACU events. Our ML model successfully identified high-risk patients, enabling proactive interventions that have the potential to decrease unplanned care. In the literature, the proportion of such preventable events is reported to range from 35% to 67% [3-6]. Our cost analysis indicated that preventing events could result in savings of approximately US $31 million over 6 years (assuming 2177 patients per year), with a positive ROI observed in the first year of implementation. Our sensitivity analyses demonstrated that the intervention achieved cost savings from prevention rates at 27%, with higher-performance scenarios yielding immediate returns. These findings underscore that even moderately effective clinical decision support tools can result in substantial cost savings when targeted to high-risk populations. Future implementations should include continuous performance monitoring to ensure that projected prevention rates are achieved. The estimated reduction in ACU events can translate to a meaningful decrease in hospital days and patient morbidity beyond financial savings. Our study targeted earlier cancer treatment stages, particularly chemotherapy, emphasizing the prevention of avoidable hospital admissions and emergency department visits. This approach not only enhances cost efficiency but also underscores the value of integrating predictive models into health care operations to improve patient outcomes. Over a 6-year period, the predictive model reduced projected costs by approximately US $31.11 million compared to the baseline. In the first year alone, the model yielded estimated savings of approximately US $910,000, increasing to US $9.46 million in year 6. The US $31.11 million figure represents the total avoided expenditure over 6 years when comparing the prediction model to standard care. These projections are based on an annual cohort of 2177 patients and an average ACU cost of US $17,031.92.

### Precision Oncology

Traditional approaches to ACU risk often rely on reactive care, addressing complications only after they occur. In contrast, our model supports a preventative approach by identifying high-risk patients before chemotherapy begins. This enables clinicians to proactively adjust treatment plans, such as dose modifications or enhanced supportive care, aligning interventions with patient-specific vulnerabilities. Model projections suggest that proactive triage informed by our risk scores could prevent ACU events in approximately 2499 patients over 6 years. As oncology shifts toward value-based care, tools that enable early, risk-based intervention while preserving operational efficiency are critical for sustainable implementation [1,2].

### Implementation Considerations

Successful implementation of predictive models requires careful attention to both clinical integration and operational feasibility. The economic viability of implementing ML algorithms in clinical workflows depends on seamless clinical integration, recommendation-action pairing, clinical oversight, and effective prioritization [30,31]. These costs include data preparation, model validation, integration into existing systems, staff training, and ongoing maintenance [13,24]. Our analysis includes the need for added staffing, including nurses and practice providers, to pair model recommendations with clinical actions. Upon deployment, patients flagged as high risk could be routed to case managers or nursing staff for follow-up via calls or assessments, with workflows integrated into existing care management protocols. While this requires training and coordination, it aligns with current practices in outpatient oncology. Even accounting for these resources, the model delivers a strong ROI. Furthermore, if higher prevention rates up to 67% are realized, as suggested by other studies [6], the resulting cost savings could be even more substantial. Overall, predictive models represent a strategic approach to reducing ACU events while improving both financial and clinical outcomes.

### Cost Model Limitations

Our study has several important limitations regarding cost estimation. First, while Medicare fee schedules (MPFS and ASP) provide standardized benchmarks, they cannot capture real-world billing complexities such as payer-specific contractual adjustments below Medicare levels (typically 15%-40% below Medicare rates) [32] or variable patient cost sharing [33]. Second, the single-center design misses undocumented out-of-network ACU events. This may underestimate ACU rates and the associated cost savings. In addition, while this CCC reflects the environment of many other cancer centers, the generalizability of our findings to other health care systems may be limited. Third, unmodeled implementation costs (eg, workforce training and workflow changes) may reduce net savings. While our ROI estimates remained positive in sensitivity analyses, real-world returns could be modestly lower. These trade-offs were necessary to enable standardized cross-center comparisons, but future work should incorporate multipayer claims data to improve precision. Fourth, specific medical costs were not available for this study; therefore, we extrapolated expenses using MPFS and ASP values from the CMS, primarily relying on Medicare fees. Fifth, ROI estimates, as shown in the one-way sensitivity analysis, are sensitive to the assumed prevention rate. If the true prevention rate is lower than modeled, the ROI may be overestimated, highlighting the importance of validation in real-world settings. Finally, we did not explicitly model all potential operational challenges, such as hiring, training, and compliance-related overhead. These unaccounted-for costs may reduce net savings in practice and should be evaluated in future implementation studies.

### Conclusions

In conclusion, this study reinforces the substantial financial burden associated with ACU in oncology patients, with marked cost differences between those who experience ACU and those who do not. By identifying high-risk patients, our model supports timely interventions that lower avoidable ACU costs and improve outcomes. These findings support the integration of predictive analytics into clinical workflows to optimize resource use, reduce preventable ACU events, and improve patient outcomes. As health care systems evolve, data-driven strategies such as ours may play a pivotal role in enhancing the efficiency and quality of cancer care.Use of Generative AI

## Supplementary material

10.2196/77891 Multimedia Appendix 1 Additional methods, cohort characteristics, and extended cost and model performance results.

## Abbreviations

ACU: acute care use
APP: advanced practice provider
ASP: average sales price
CCC: comprehensive cancer center
CCPD: cost of care per patient per day
CMS: Centers for Medicare and Medicaid Services
CPT: Current Procedural Terminology
EHR: electronic health record
FTE: full-time equivalent
HCPCS: Healthcare Common Procedure Coding System
ML: machine learning
MPFS: Medicare Physician Fee Schedule
ROI: return-on-investment
TCPP: total cost per patient per period
