# Supplementary material for: Cost-Benefit Analysis of Preventing Acute Care Use in Oncology Patients Following Systemic Therapy Using Medicare Claims Data: Retrospective Cohort Study
Source: JMIR Med Inform. 2025 Dec 11;13:e77891. doi: 10.2196/77891 (PMC12698071; doi:10.2196/77891)
Supplement: Multimedia Appendix 1 [file medinform-v13-e77891-s001.doc]

# Appendix

## General Information

### Data Sharing Statement

The clinical data is sourced from the Stanford Health Care Alliance (SHA), encompassing records from Stanford Health Care (SHC), Valley Care Hospital (ValleyCare), and the University Healthcare Alliance (UHA). Due to privacy and ethical considerations, these data are not publicly accessible. However, disidentified data may be provided to researchers meeting the necessary criteria for confidential data access. The cost extrapolation data can be accessed via CMS.org.

### Further Methods and Materials

#### Cost calculation in detail

Both the Medicare Physician Fee Schedule (MPFS) [1] and Average Sales Price (ASP) [2] calculation followed the CMS method [3] and earlier research [4,5]. MPFS are discerned using (1) relative value units (RVU), which are assigned to services; (2) a geographic practice cost index (GPCI), which adjusts RVUs for geographic cost differences; and (3) a conversion factor (CF), which is dollar amount accounting for inflation and economic factors, converting RVU values into US $ amounts. Both RVU and the GPCI come with three components and are suffixed accordingly. MPFS fees for an HCPCS code *c* each year *x* and quarter *q*:


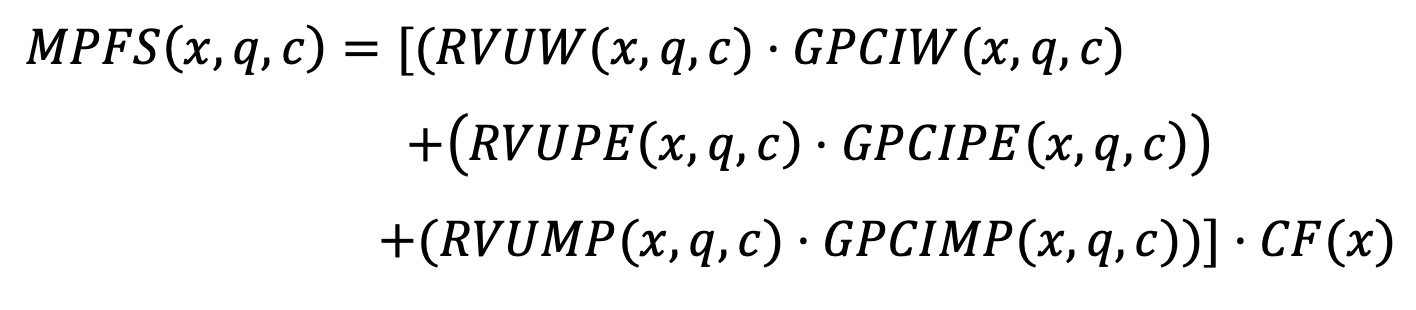
 (1)

ASP is established using the maximum payment limit (PL), which is set at 106% of the average sales price. ASP fees for a HCPCS code *c* each year *x* and quarter *q* are stipulated as follows:


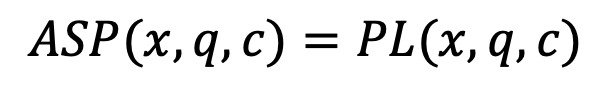
 (2)

We combined ASP and MPFS fees into one variable called ‘combined fee’, summing values with the same HCPCS code *c* each year *x* and quarter *q* as:


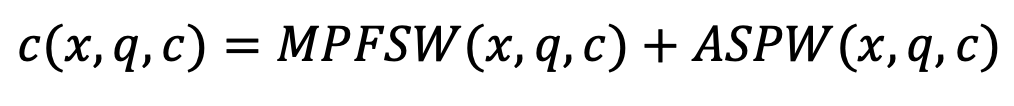
 (3)

CPT and HCPCS codes were grouped using CMS categories. We focused on Medicare-allowable costs for professional services and drugs. Other cost components like facility fees or patient cost-sharing were not included. We sum all fees incurred by n patients over t days and then divide this total by the total number of patient-days (
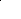
) to obtain the average cost per patient per day. These daily average costs are then aggregated over the entire therapy period to yield TCPP, which represents the sum of all fees incurred during the given period of therapy (see Table S1). In more detail, we have an outer summation over all values j from 1 to n and an inner summation over all values i from 1 to t. We receive a TCCP for a specific day and patient.


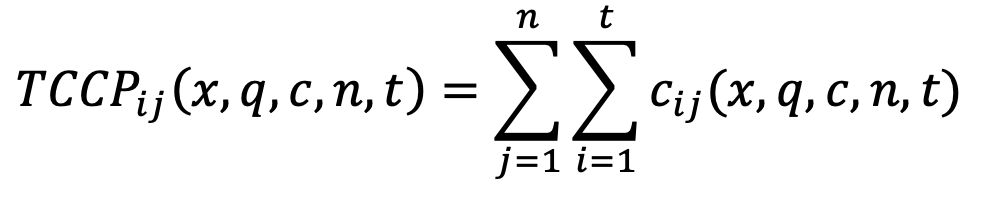
 (4)

To compare values between ACU and Non-ACU, we update outer summation to be values j from 1 to n_group_ and we divide value by total number of n_group_ to receive an average TCCP per patient group, either ACU or Non-ACU.


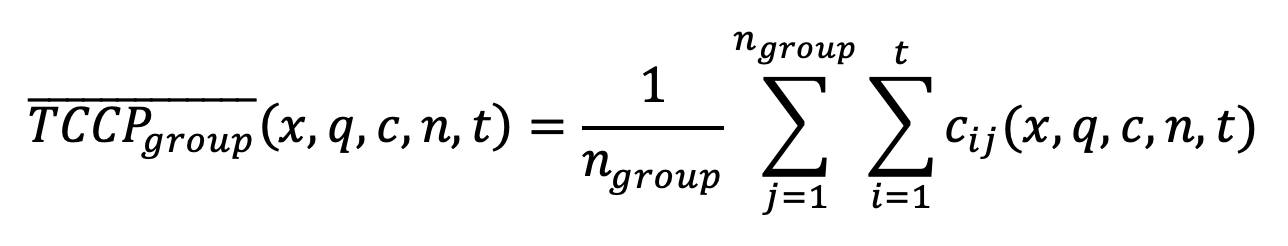
 (5)

The CCPD is computed by taking TCCP and dividing it by length of period of interest ∆t. We receive a CCPD for a specific day and patient (Table S2).


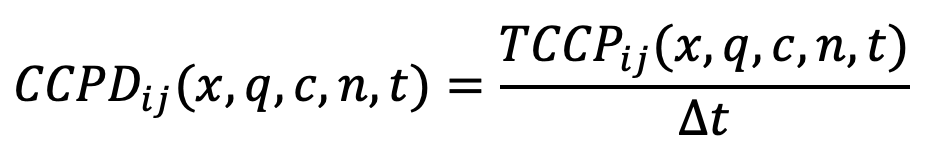
 (6)

To compare between ACU and Non-ACU groups, we divide average TCCP per patient group by number of days within the day period, which gives us the average CCPD per patient group.


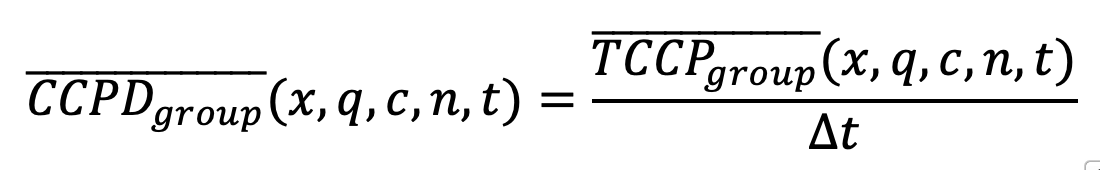
 (7)

| TCPP | Total Cost per Patient per Period | All costs on each day summed till end of period per patient | 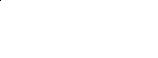 |
| --- | --- | --- | --- |
| CCPD | Cost of Care per Patient per Day | TCPP per patient is divided by the number of days within the period length | 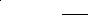 |

Table S1. Definitions of Cost Variables used for modelling (US dollars).

#### Cost models in detail

This section details the cost-benefit analysis [6] conducted on total expense of ACU with and without a prediction model. A similar study developed a statistical classifier to predict 30-day readmission rates [7]. We used constants from the literature, inferred fee values, and model parameters from the prediction model [8]. The value of US $1 million *C_deploy_* and annual maintenance cost of US $200,000 *C_annual_* were based on values found in literature, a use case, and internal estimations [9-11].

The total cost without using a prediction model *C_0_* was estimated using the number of patients, *N_acu,0_*, prevalence of ACU cases *p_acu_,* and expense per ACU case *C_acu_ for a year x.* The number of ACU patients *N_acu,0_* is estimated by multiplying the *p_prev_* with the total number of patients *N_total_.*


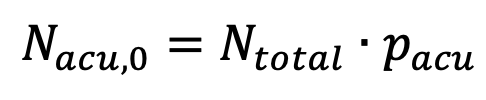
 (8)

Total cost estimation without prediction model:


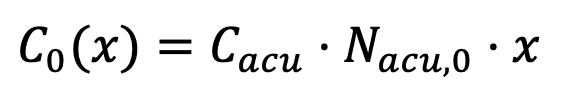
 (9)

The total cost with a model *C_1_* was computed by considering deployment cost *C_deploy_*, annual maintenance expense *C_annual_*, cost for remaining ACU cases *C_acu,1_*, expenditure of additional nurses, *C_nurse_,* and extra APPs *C_app_*. To extrapolate *C_acu,1_*, we need to find the number of ACU cases predicted *N_acu,1_*, for which we require the number of preventable ACU cases *N_prev,acu_* and from that number of patients correctly predicted *N_true,pos_*. We find the number of cases that were forecasted as positive and are negative *N_false,pos_*. For this, first we compute the number of patients not having an ACU *N_noacu,0._*


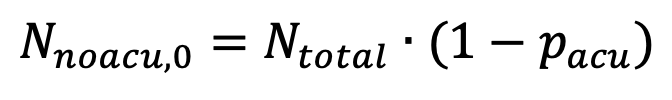
 (10)


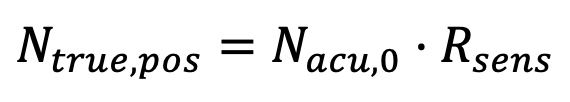
 (11)


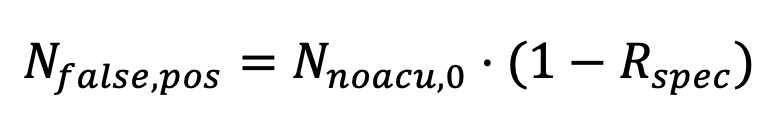
 (12)


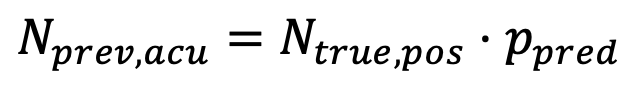
 (13)


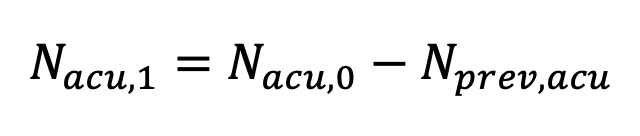
 (14)


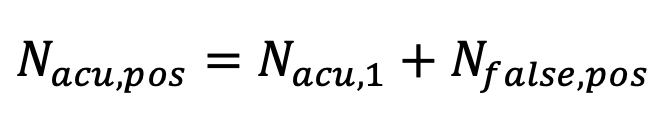
 (15)

Now, we estimate the total cost for remaining ACU cases after prevention *C_acu,1_*:


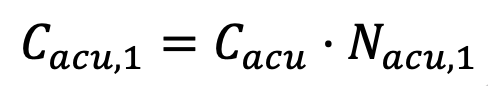
 (16)

Now we assess the staff terms *C_nurse_* and *C_app_*:

*
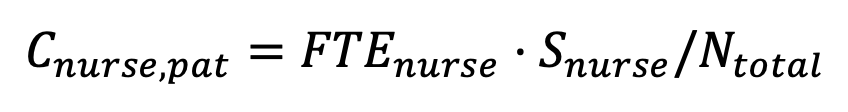
* (17)

*
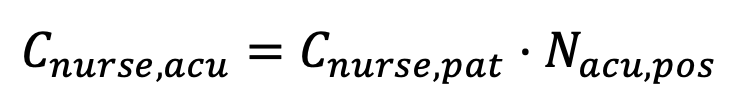
* (18)

*
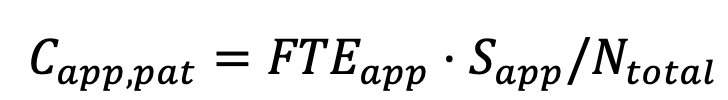
* (19)

*
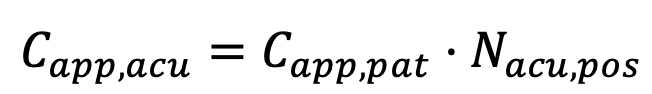
* (20)

Total cost calculation with prediction model *C_1_*:

*
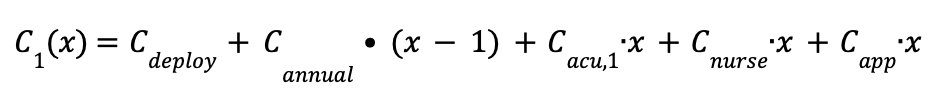
* (21)

### Implementation Workflow

As described in the manuscript, we expect our intervention to prevent unnecessary ACU events in the depicted way by preventing unnecessary ACUs caused by the therapy cycle (S10).


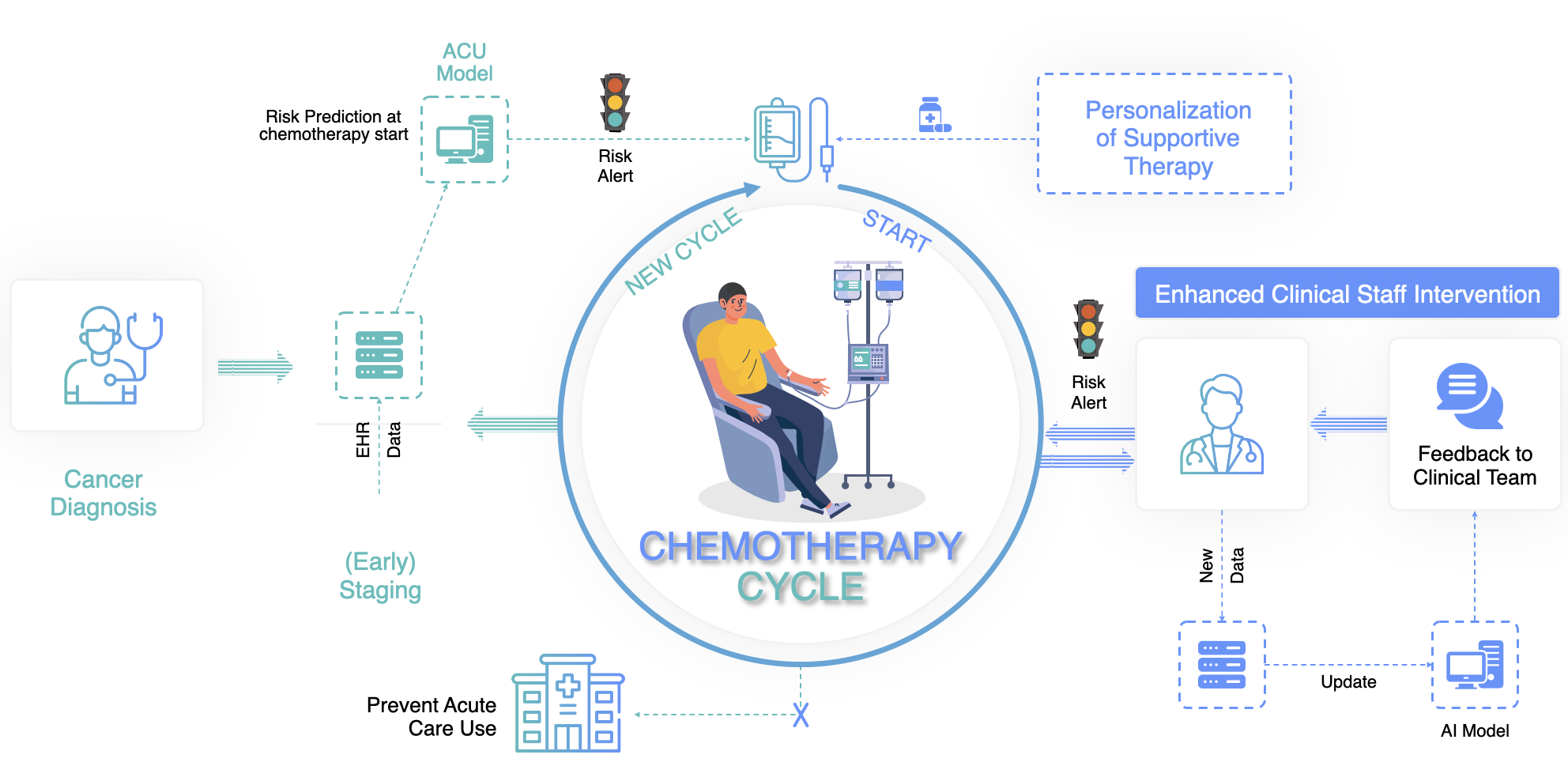


Fig. S2. Workflow integration schema. This diagram depicts the workflow we expect to happen once we deploy the ML model at a hospital. It shows on one hand what necessary involvement is expected and on the other how this involvement is expected to reduce ACU involvement.

## Further Results

### Cohort in detail

Summary of baseline characteristics for the total cohort (N=20,556), stratified by patient group: Non-ACU and ACU. Patient demographics, tumor stages, and tumor types are included, with statistically significant differences marked, as seen in Table S3.

| **Characteristic** | **Description** | **Total** | **Non-ACU** | **ACU** | **p Value** |
| --- | --- | --- | --- | --- | --- |
| Patient Count | Total Unique Patients, No. (%) | 20,556 (100) | 16,736 (81) | 3,820 (19) | - |
| Gender | F ^c^, No. (%) | 10,914 (53) | 9,022 (83) | 1,892 (17) | 0.000 |
|  | M ^c^, No. (%) | 8,904 (43) | 7,092 (80) | 1,812 (20) | 0.000 |
| Ethnicity | Latino ^c^, No. (%) | 2,606 (13) | 1,993 (76) | 613 (24) | 0.000 |
|  | Non-Hispanic/Latino ^c^, No. (%) | 17,212 (84) | 14,121 (82) | 3,091 (18) | 0.000 |
| Race | Asian, No. (%) | 4,391 (21) | 3,496 (80) | 895 (20) | 0.0530 |
|  | Black, No. (%) | 532 (3) | 409 (77) | 123 (23) | 0.345 |
|  | Hawaiian Or Pacific, No. (%) | 212 (1) | 154 (73) | 58 (27) | 0.117 |
|  | Other Race ^c^, No. (%) | 3,287 (16) | 2,514 (76) | 773 (24) | 0.000 |
|  | White ^c^, No. (%) | 11,321 (55) | 9,478 (84) | 1,843 (16) | 0.000 |
| Insurances | Medicaid ^c^, No. (%) | 2,699 (13) | 1,907 (71) | 792 (29) | 0.000 |
|  | Medicare, No. (%) | 7,303 (36) | 6,047 (83) | 1,256 (17) | 0.360 |
|  | Other Insurance ^c^, No. (%) | 384 (2) | 310 (81) | 74 (19) | 0.000 |
|  | Private Insurance ^c^, No. (%) | 9,432 (46) | 7,850 (83) | 1,582 (17) | 0.360 |
| Tumor Stage | 0 ^c^, No. (%) | 508 (3) | 471 (93) | 37 (7) | 0.000 |
|  | 1 ^c^, No. (%) | 2,513 (12) | 2,189 (87) | 324 (13) | 0.001 |
|  | 2 ^c^, No. (%) | 2,456 (12) | 2,058 (84) | 398 (16) | 0.000 |
|  | 3 ^c^, No. (%) | 1,311 (6) | 1,023 (78) | 288 (22) | 0.000 |
|  | 4 ^c^, No. (%) | 2,363 (12) | 1,644 (70) | 719 (30) | 0.000 |
| Tumor Type | Breast ^c^, No. (%) | 5,073 (25) | 4,579 (90) | 494 (10) | 0.000 |
|  | Gastrointestinal Upper ^c^, No. (%) | 1,102 (5) | 868 (79) | 234 (21) | 0.000 |
|  | Genitourinary, No. (%) | 572 (3) | 374 (65) | 198 (35) | 0.128 |
|  | Gynecologic ^c^, No. (%) | 1,596 (8) | 1,320 (83) | 276 (17) | 0.000 |
|  | Head Neck ^b^, No. (%) | 1,372 (7) | 1,013 (74) | 359 (26) | 0.003 |
|  | Hepatobiliary, No. (%) | 1,453 (7) | 1,150 (79) | 303 (21) | 0.181 |
|  | Lung Neurologic ^b^, No. (%) | 490 (2) | 404 (82) | 86 (18) | 0.005 |
|  | Lung Thoracic ^a^, No. (%) | 1,541 (8) | 1,153 (75) | 388 (25) | 0.013 |
|  | Lymph, No. (%) | 1,600 (8) | 1,156 (72) | 444 (28) | 0.101 |
|  | Pancreas ^c^, No. (%) | 720 (4) | 483 (67) | 237 (33) | 0.000 |
|  | Prostate ^c^, No. (%) | 1,635 (8) | 1,500 (92) | 135 (8) | 0.000 |
|  | Sarcoma ^c^, No. (%) | 560 (3) | 374 (67) | 186 (33) | 0.000 |
|  | Skin ^c^, No. (%) | 814 (4) | 714 (88) | 100 (12) | 0.000 |

Table S3. Characteristics of cohort by patient group. Superscripts indicate significance based on chi-square tests: *P*≤.05: a, *P*≤.01: b, *P*≤.001: c. Missing values excluded and may result in percentages not summing to 100%.

### Cost in detail

As seen in table S3, at all-time points (30, 90, and 180 days), the ACU group incurred significantly higher TCPP than the Non-ACU group, with differences widening over time (e.g., US $17,030.92 vs. US $9,591.06 at 180 days). Similarly, the CCPD was consistently higher in the ACU group, though both groups experienced a decline over time (e.g., US $94.62 vs. US $53.28 at 180 days). These differences were statistically significant (*P*<.001) for all the annotated timepoints in Figure 2. There is more information provided on the standard deviations in Table S1.

In Figure S4, the distribution of CCPD on day 180 shows a significant difference between the ACU and Non-ACU groups, with the ACU group having a much higher mean CCPD (*P*<.001). For clarity, values above US $3,000 were excluded (Fig. S4 A).

| **Group** | **Value** | **30 days** | **90 days** | **180 days** |
| --- | --- | --- | --- | --- |
| Non-  ACU | Average TCPP | 3,211.55 ^c^ (± 4,692.06, (3,140.45, 3,282.64)) | 6,675.61 ^c^ (± 8,135.89, (6,552.34, 6,798.88)) | **9,591.06** ^c^ (± 10,785.83, (9,427.64, 9,754.48)) |
|  | Average CCPD | 107.05 ^c^ (± 156.40,  (104.68, 109.42)) | 74.17 ^c^ (± 90.40, (72.80, 75.54)) | **53.28** ^c^ (± 59.92, (52.39, 54.19)) |
| ACU | Average TCPP | 5,237.36 ^c^ (± 4,865.17, (5,083.03, 5,391.69)) | 11,240.87 ^c^ (± 9180.60, (10949.65, 11532.09)) | **17,030.92** ^c^ (± 13,056.63, (16616.74, 17445.09)) |
|  | Average CCPD | 174.58 ^c^ (± 162.17, (169.43, 179.72)) | 124.90 ^c^ (± 102.01, (121.66, 128.13)) | **94.62** ^c^ (± 72.54, (92.32, 96.92)) |

Table S4. TCPP and CCPD by group over time. Values are presented as mean ± SD, (CI 95%), in US dollars. Superscripts indicate significance of between-groups comparisons based on t-tests: *P*≤.05: a, *P*≤.01: b, *P*≤.001: c)

In Figure S5 we see the distribution of the total costs on day 180. There is a clear difference in total cost between the groups. The means are significantly different (*P*<.001) (Fig. S5). Since the distribution of the patient groups looks the same, it was omitted in this graph. This suggests that the overall distributions of the patient groups are similar, implying that significant cost differences are not due to differences in size or distribution but rather to true cost differences.


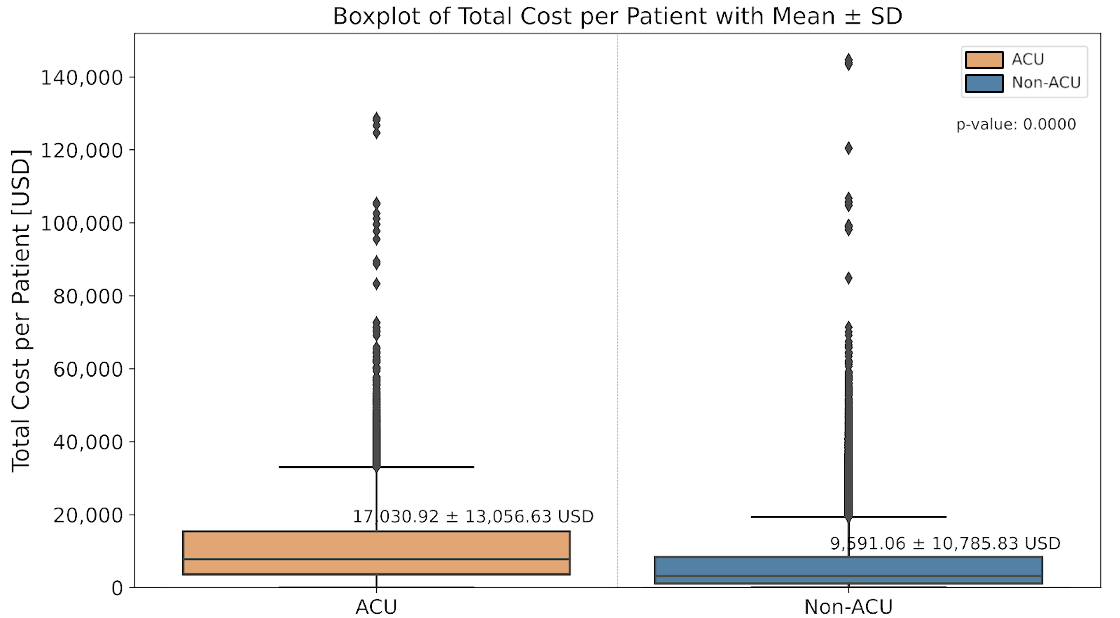


Figure S5. Box plots of total costs at day 180 stratified by ACU status. ACU group: n = 3,820; Non-ACU group: n = 16,736.

Figure S6 B presents a histogram showing the count of patients in each group, highlighting that while there are more Non-ACU patients, there is still a notable cost difference between the two groups. The histogram shows the distribution of CCPD across the two groups. We can infer that a larger proportion of high-cost patients are in the ACU group, suggesting that the cohort with higher costs includes a larger share of ACU patients. The extreme p-value (*P*<.001) reflects a near-certain difference between the groups under the test’s assumptions (Fig. S4 A).


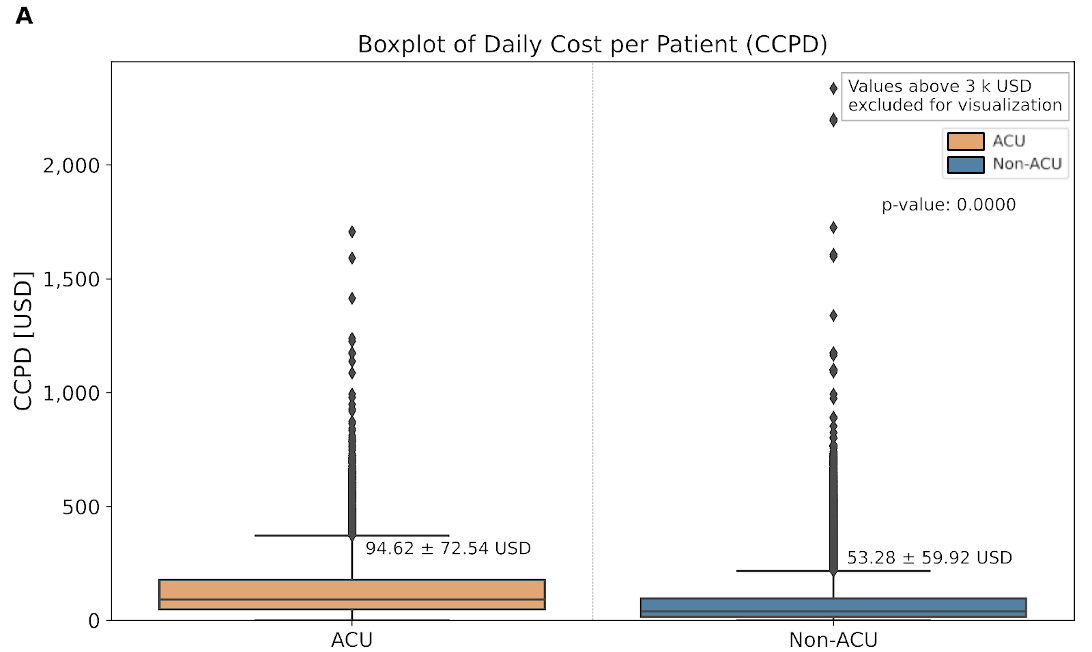


**
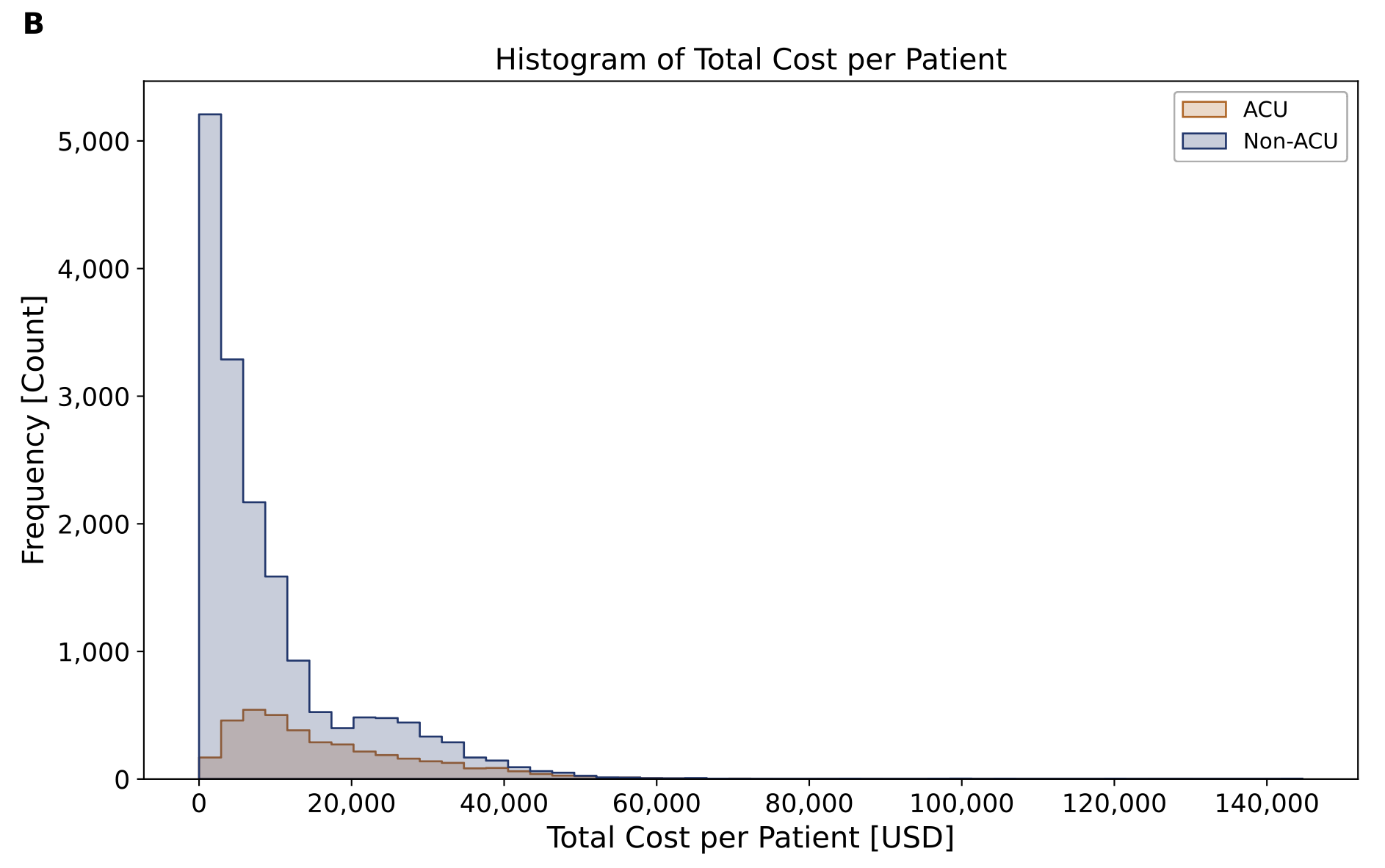
**

Figure S6. (A) Box plots of CCPD since the start of systemic therapy, stratified by patient groups, (B) Histogram of CCPD count stratified by patient groups. ACU gorup: n = 3,820; orange and Non-ACU group: n = 16,736; blue.

### ML Models

These scores we received in our model training. We trained three model types, each with and without weighting, like existing methods [8]. Their scores can be found in the table S7 [8].


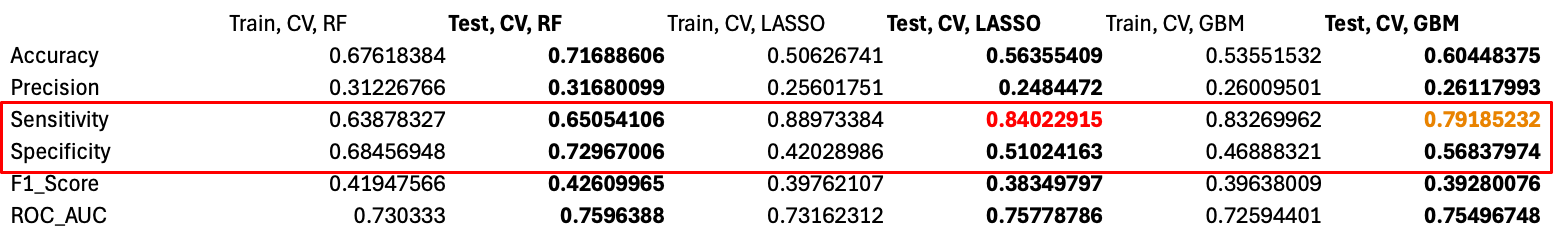


Table S7. Score Results from Model Training, (Split: [2010, 2018], [2019, 2022]).

To validate our cost model, we analyzed break-even points for sensitivity *R_sens_*, number of ACU patients *N_total_*, and prevalence of ACU while keeping the other parameters constant. A ROI was achieved with roughly *R_sens_* ≥ 0.46 (assuming *R_spec_* of 0.50), *N_total_* ≥ 1,300 and *p_prev_* ≥ 20%. Lower values were not considered meaningful for the model's application. Even with a lower sensitivity of 0.79 and a specificity of 0.54 from the GBM model with parameter weighting, cost savings remained large: US $792 thousand in the first year and US $8.75 million by the sixth year, assuming other parameters stay constant. This amounts to a total of US $28.63 million in savings over the first six years. For both model parameters, statistical analyses show significant cost differences between the models with and without prediction. The number of patients in treatment *N_total_* is the most impactful parameter, with sensitivity *R_sens_* and prevalence of ACU *p_prev_* are relevant, but less influential on cost reduction at lower values. The maximal reduction has a t-statistic of 4.009 and a significant p-value (*P*<.01). This suggests a significant difference between the means of results from the C_0_ and C_1_ models.

### Services

In Figure S8 one can see that there is both a higher mean of service count and higher mean of service fees attributed to the ACU patients. In Figure S8A, we can see that the mean count of services per patient varies per patient group. The x-axis looks at the different code descriptions that the codes of a service belong to. For example, Pathology and Laboratory Procedures the mean number of services of an ACU patient is much higher, more than three times the mean of a non-ACU patient, while Radiology Procedures seem to have a more similar count for both patient groups. Values above 800 have been excluded for visualization purposes. All top 5 most common codes show a significant difference in means between the groups. In Figure S8B, we can see a similar visualization for the cost of these services. The costs related to Medicine Services and Procedures are two times higher for ACU patients than non-ACU patients, while the numbers for Drugs Administered Other than Oral Method are around the same. For visualization purposes values above US $40,000 were excluded. All top 5 most expensive codes show a significant difference in means between the groups. Furthermore, a Mann-Whitney test delivered p values *P*<.001 implying a difference in mean between the ACU and Non-ACU group in terms of count and cost for a service. In Figure S9 one can see that there is a higher percentage of service codes attributed to the ACU patients. This suggests that ACU patients are receiving a higher amount of therapy services, due to the added ACU events. A t-test to compare the number of services per ACU group showed that there is a significant difference between ACU status and days since chemo in terms of service code frequencies. It resulted in a t-test value of 79,538.668 and a *P*<.001.


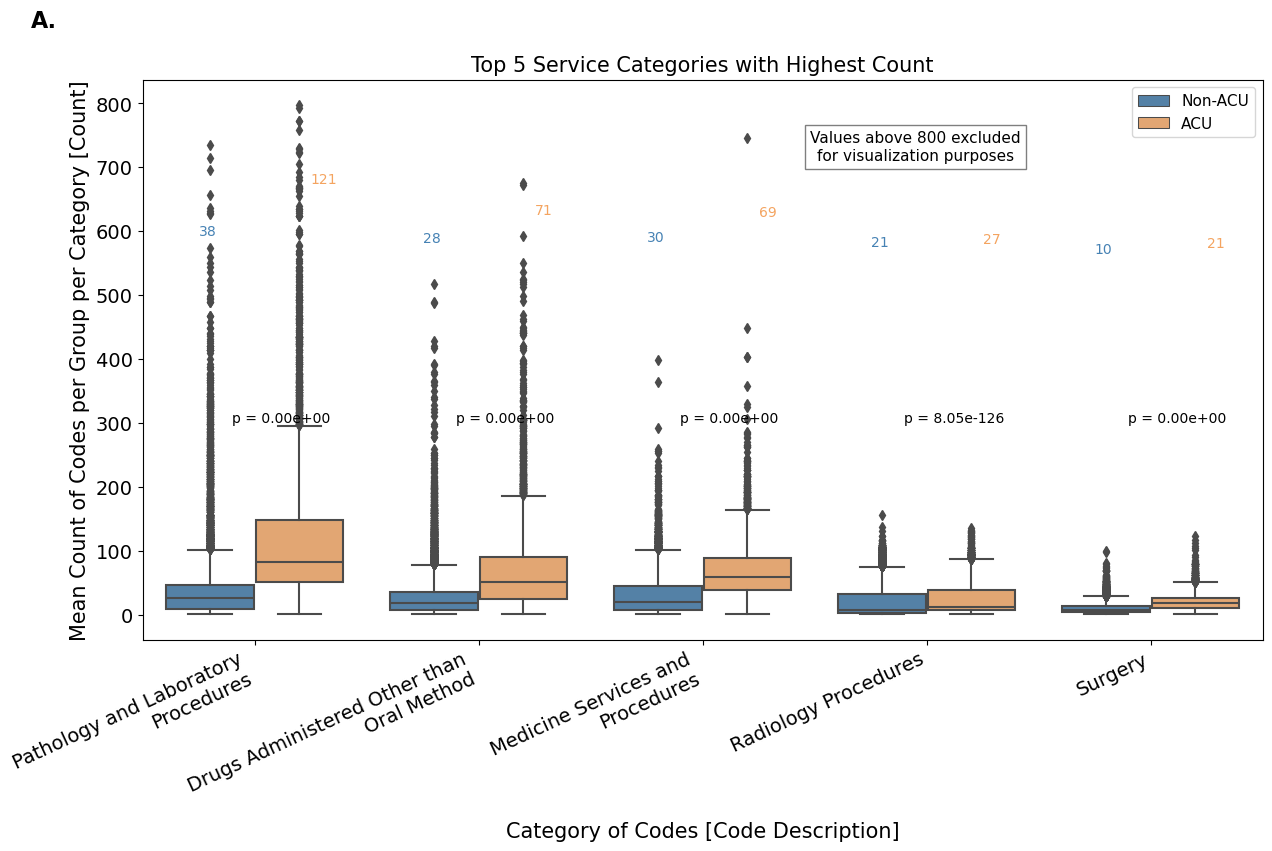

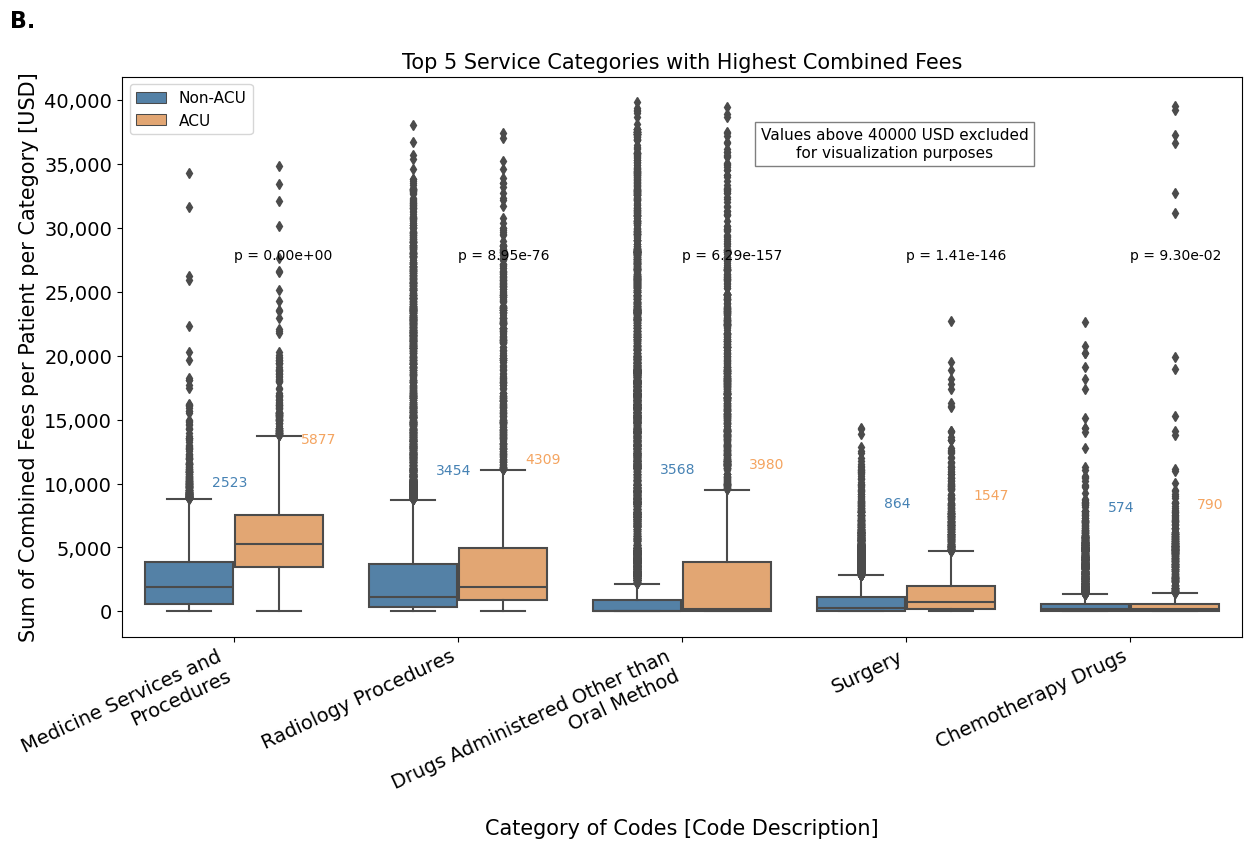


Figure S8. Top 5 most charged and costly categories by groups (A) Total count per category. ACU group: n = 3,820; orange and Non-ACU group: n = 16,736; blue. (B) Total costs per category. ACU group: n = 3,820; orange and Non-ACU group: n = 16,736; blue.


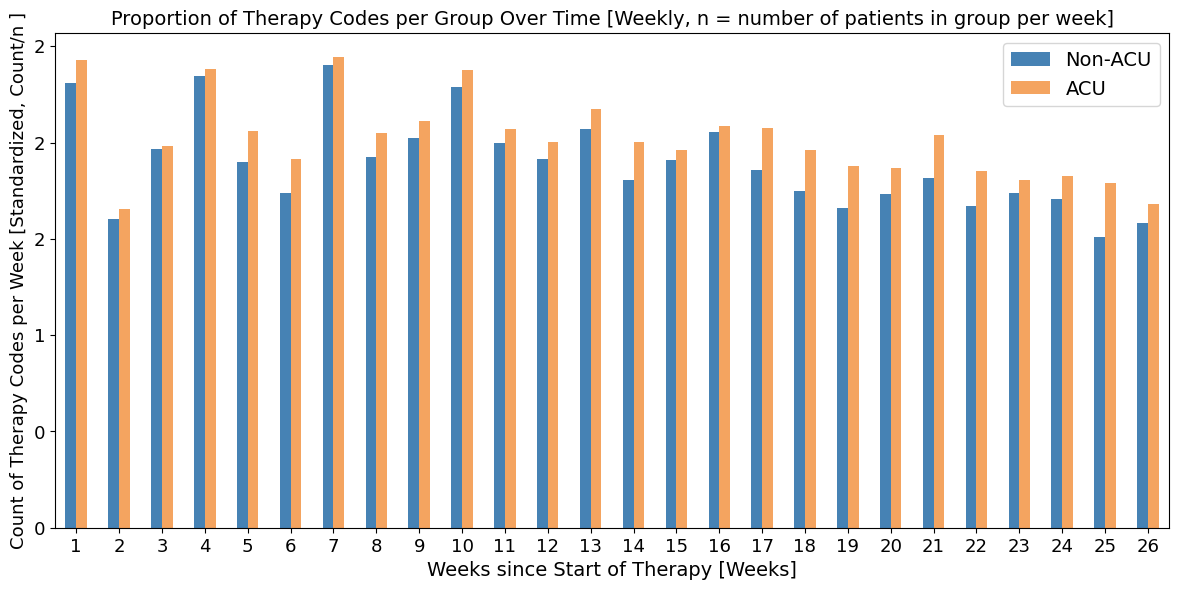


Figure S9. Percentage of standardized count of therapy codes stratified by group. ACU group: n = 3,820; orange and Non-ACU group: n = 16,736; blue.

### Sensitivity Analysis

Figure S10 below summarizes the relationship between the prevention rate (p_pred_) and the break-even year. A vertical reference line marks the base-case value p_pred_ =35, while a horizontal dashed line represents the six-year evaluation window. This visualization demonstrates the minimum threshold of model effectiveness needed for viable return on investment.


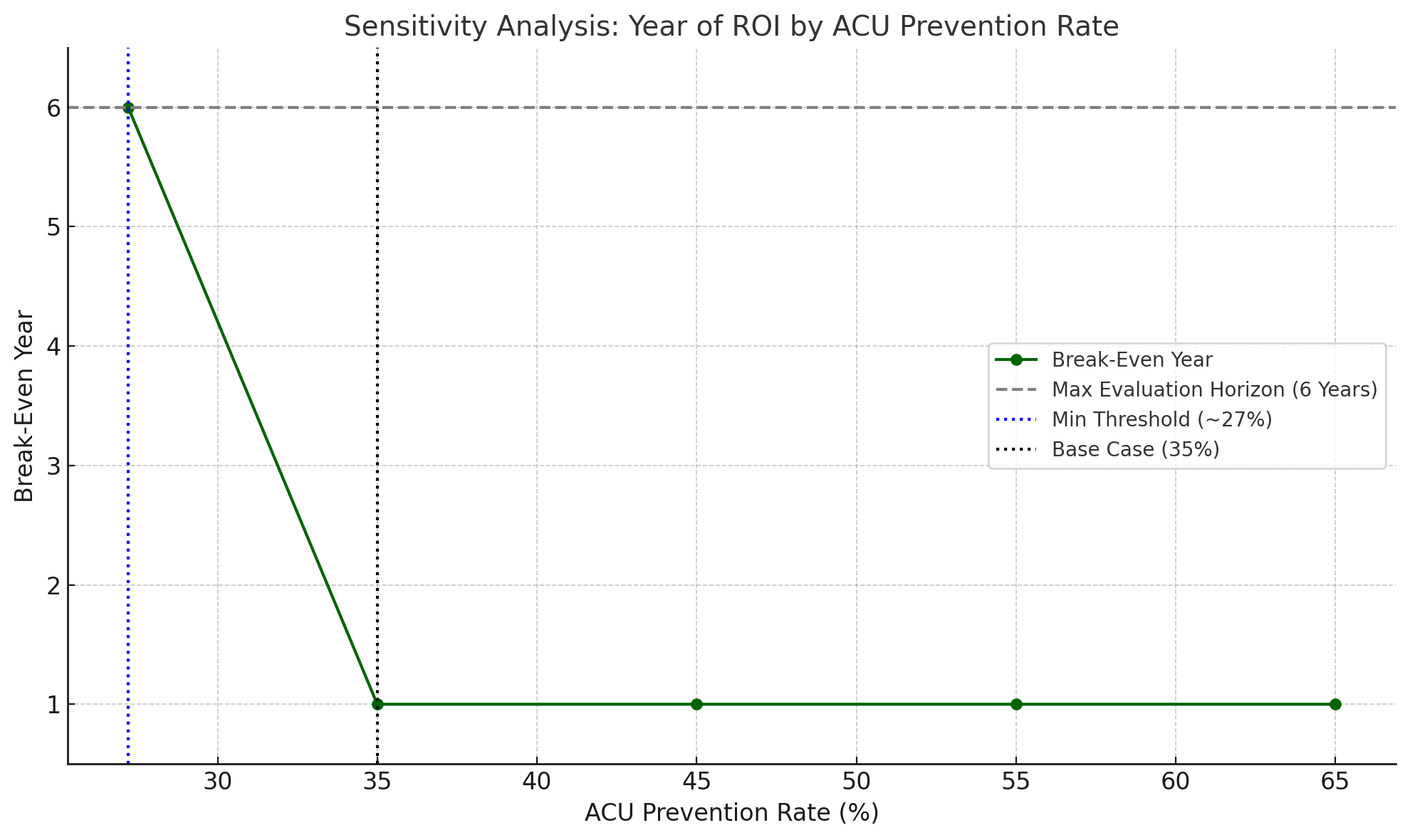


Fig. S10. Break-Even Year by ACU Prevention Rate. The first year in which net savings become positive is shown for each value of p_pred_. A prevention rate of at least 27% is required to break even within 6 years. The vertical dotted line marks the base-case assumption of p_pred_ =35%, which achieves break-even in Year 1.

**References**

1. Centers for Medicare & Medicaid Services. Physician Fee Schedule. CMS.gov. July 12, 2024. Accessed August 2, 2024. https://www.cms.gov/medicare/payment/fee-schedules/physician

2. Centers for Medicare & Medicaid Services. Average Sales Price (ASP) Reporting. CMS.gov. July 23, 2024. Accessed August 2, 2024. https://www.cms.gov/medicare/payment/part-b-drugs/asp-reporting

3. Centers for Medicare & Medicaid Services. *Medicare Payment Systems Educational Tool*.; 2024. Accessed August 2, 2024. https://www.cms.gov/Outreach-and-Education/Medicare-Learning-Network-MLN/MLNProducts/html/medicare-payment-systems.html#Hospital

4. Blayney DW, Seto T, Hoang N, Lindquist C, Kurian AW. Benchmark Method for Cost Computations Across Health Care Systems: Cost of Care per Patient per Day in Breast Cancer Care. *JCO Oncol Pract*. 2021;17(10):e1403-e1412. doi:10.1200/OP.20.00462

5. Esposito T, Reed R, Adams RC, Fakhry S, Carey D, Crandall ML. Acute Care Surgery Billing, Coding and Documentation Series Part 1: Basic Evaluation and Management (E/M), Emergency Department E/M, Prolonged Services, Adult Critical Care Documentation and Coding. *Trauma Surg Acute Care Open*. 2020;5(1):e000578. doi:10.1136/tsaco-2020-000578

6. Centers for Disease Control and Prevention, Office of Policy, Performance, and Evaluation. Cost-Benefit Analysis. CDC Polaris Economic Evaluation. October 20, 2021. Accessed August 2, 2024. https://www.cdc.gov/policy/polaris/economics/cost-benefit/index.html#:~:text=What%20is%20cost%2Dbenefit%20analysis,are%20expressed%20in%20monetary%20units.&text=Both%20CBA%20and%20cost%2Deffectiveness,(CEA)%20include%20health%20outcomes.

7. Bayati M, Braverman M, Gillam M, et al. Data-Driven Decisions for Reducing Readmissions for Heart Failure: General Methodology and Case Study. *PLOS ONE*. 2014;9(10):e109264. doi:10.1371/journal.pone.0109264

8. Peterson DJ, Ostberg NP, Blayney DW, Brooks JD, Hernandez-Boussard T. Machine Learning Applied to Electronic Health Records: Identification of Chemotherapy Patients at High Risk for Preventable Emergency Department Visits and Hospital Admissions. *JCO Clin Cancer Inform*. 2021;5:CCI.21.00116. doi:10.1200/CCI.21.00116

9. Morse KE, Bagley SC, Shah NH. Estimate the hidden deployment cost of predictive models to improve patient care. *Nat Med*. 2020;26(1):18-19. doi:10.1038/s41591-019-0651-8

10. Coop R. What is the Cost to Deploy and Maintain a Machine Learning Model? phData. May 20, 2021. Accessed August 2, 2024. https://www.phdata.io/blog/what-is-the-cost-to-deploy-and-maintain-a-machine-learning-model/

11. Zimelewicz E, Kalinowski M, Mendez D, et al. ML-Enabled Systems Model Deployment and Monitoring: Status Quo and Problems. In: Bludau P, Ramler R, Winkler D, Bergsmann J, eds. *Software Quality as a Foundation for Security*. Springer Nature Switzerland; 2024:112-131. doi:10.1007/978-3-031-56281-5_7
